# What Do We Know about Suicide Bereavement, and What We Can Do to Help Suicide-Loss Survivors?

**DOI:** 10.3390/ijerph20085577

**Published:** 2023-04-19

**Authors:** Yossi Levi-Belz, Karolina Krysinska, Karl Andriessen

**Affiliations:** 1 The Lior Tsfaty Center for Suicide and Mental Pain Studies, Ruppin Academic Center, Emek Hefer 40250, Israel; 2 Centre for Mental Health, Melbourne School of Population and Global Health, The University of Melbourne, Parkville, VIC 3010, Australia

“*Suicide is not only the end of life for the deceased but also the beginning of a highly challenging life for those left behind*”. While most efforts and scientific studies concerning suicide focus on prevention [1], these touching words of Edwin Shneidman remind us that the societal toll of suicide reaches well beyond human loss. Recent studies [2,3,4] noted that each suicide affects, on average, five family members and up to 135 community members. Considering that approximately 700,000 people worldwide die by suicide annually [5], estimates are that 60 million people are added to the suicide-loss survivors’ population each year. These numbers alone underscore the importance of understanding how we can help those bereaved by suicide in their psychosocial journey of dealing with their grief.

A large body of data has found that suicide-loss survivors are at greater risk than other bereaved individuals and the general population for many severe psychological and health problems. Research has revealed that suicide-loss survivors present higher levels of depression, and suicidal ideation and behavior [6,7] than other bereaved individuals. Moreover, those bereaved by suicide can experience adverse grief reactions such as prolonged grief disorder symptoms [8,9] which can persist even several years following the loss.

Suicide loss has also been associated with other deleterious psychosocial consequences. The turmoil of guilt, shame, anger, and embarrassment that follow the suicide of a loved one (e.g., [10]) seems to facilitate social withdrawal and efforts to conceal the cause of the significant other’s death [11,12]. These behaviors, along with low self-disclosure [13], low perceived social support [14], and high levels of thwarted belongingness [15], have all been found to characterize suicide-loss survivors. Together, these features can be viewed as a call to action to devise ways to help those bereaved by suicide.

We have co-edited a Special Issue which focuses on suicide bereavement and postvention, with the aim to highlight the current knowledge and advances in the research, practices, and policies regarding suicide bereavement. The Special Issue includes 19 papers which cover a wide range of topics, including (a) the impact of suicide on individuals and communities, and adverse grief reactions, (b) the impact of suicide on health professionals, and (c) coping and support for those bereaved by suicide. The findings of these studies are an important step forward in our understanding of additional approaches to support those bereaved by suicide in their healing journey.

## 1. Impact of Suicide and Adverse Grief Reactions

Macdonald and colleagues [16] conducted a qualitative study with participants who had lost a male family member by suicide. The participants reported on their perception of the suicide and their struggles with meaning making and finding closure. Mathieu and colleagues [17] compared bereavement after different modes of death six months after the loss and did not find a difference in coping styles. However, having a previous mental health diagnosis was associated with increased avoidant and problem-focused coping, and stigma and shame were associated with increased avoidant coping. Women were more likely to report using adaptive coping than men. Goodall and colleagues [18] conducted a systematic review on continuing bonds in suicide bereavement and found that continuing bonds can be experienced in various ways and that most people bereaved by suicide tended to perceive it primarily as a positive experience that may help with coping and meaning making.

Little is known about suicide bereavement in later life, and to address this gap Hafford-Letchfield and colleagues [19] explored how suicide loss can intersect with ageing and what supports are needed for this population. Interestingly, several participants had guided their social network to receive the necessary support. In another important study, Tiech Fire and colleagues [20] focused on school staff, with the aim of understanding the impact of student suicides on post-trauma and grief symptoms among teachers, counsellors, and school principals. The authors found that homeroom teachers suffered more from complicated grief and post-traumatic stress disorder (PTSD) compared to psychologists and counsellors and, as such, should receive more preparatory training. Additionally, in the context of schools, Khalid and colleagues [21] found encouraging results from a training program that increased preparedness and skills in school staff to respond to student suicides. Rivant and colleagues [22] focused on suicide bereavement among ethnic minority groups. They found that this population suffers from a lack of support and high prevalence of stigma in the aftermath of suicide loss. These preliminary results broaden our understanding of a minority population which requires special attention, more visibility, and better access to support services.

Andriessen and colleagues [23] examined the experiences of adolescents who participate in qualitative research interviews about suicide-related experiences, as there is a perception of risk regarding asking young people about these topics. Importantly, the findings indicated that bereaved adolescents, as well as parents and clinicians, could safely participate in research interviews regarding their experiences of grief after suicide, and they highly valued this opportunity to share their experiences. These findings can be used to build future study designs to help us to better understand the processes of grief after suicide among adolescents.

Several studies in our Special Issue examined deleterious and long-term effects of suicide bereavement. In a latent class analysis, Grafiadeli and colleagues [24] found that about half of the examined population showed elevated prolonged grief disorder symptoms, and one-third of the sample also showed PTSD symptoms. In a six-year longitudinal study, Levi-Belz and Brinbaum [15] highlighted that suicide bereavement facilitated higher levels of depression and suicide ideation. Other studies also emphasized the moderators of these negative outcomes. Levi-Belz and Brinbaum [15] found that the sense of belongingness can serve as a buffer against depression, as suicide-loss survivors with higher levels of belongingness were less vulnerable to depression at later assessments. Levi-Belz and Ben-Yaish [9] highlighted interpersonal factors, such as social support and self-disclosure, that serve as moderators of prolonged grief symptoms following suicide bereavement. Pitman and colleagues [25] examined whether bereavement by various causes had a deterrent effect on alcohol and drug use in bereaved individuals. They found no group differences regarding reduced alcohol use, but a significantly greater proportion of people bereaved by sudden unnatural causes, including suicide, reduced/stopped drug use post-bereavement compared to people bereaved by sudden natural causes.

## 2. Impact of Suicide on Mental Health Professionals

Both health and mental health professionals are at risk of losing a client or a colleague to suicide. The integrated review of 17 articles conducted by Causer and colleagues [26] emphasized the impact that a suicide by a colleague may have on workers in healthcare and other settings. They found that the perceptions of grief are complicated by professional identities and workplace cultures. Tamworth and colleagues [27] revealed that losing a patient to suicide can be a profoundly traumatic experience for psychiatrists. Peer support was key in processing their grief. Pisnoli and Van der Hallen [28] focused on mental health professionals who had a client die by suicide. These authors examined the relationships between attitudes toward (client) suicide and the psychological impact of client suicide as perceived by the therapists. It was found that those who believe that “suicide can and should be prevented” reported a higher impact in the form of short-term emotional and long-term professional impacts. Together, these studies indicate a need for systemic postvention, enhanced peer support, and promotion of self-care among mental health professionals.

## 3. Coping and Support for Those Bereaved by Suicide

Agnietė Čepulienė and Skruibis [29] conducted a qualitative interview study and analyzed the data using reflexive thematic analysis. They found that spirituality can function as a resource after a loved one’s suicide and can even contribute to post-traumatic growth after the loss, while spirituality-related issues, such as stigmatization and a lack of personally meaningful traditions, can contribute to higher distress among suicide-loss survivors. In a scoping review, Higgins and colleagues [30] indicated the potential effectiveness of peer-led suicide bereavement support, but also highlighted a need for rigorous design and evaluation of peer-led interventions in this field. Hybholt and colleagues [31] explored the experiences of members of peer-led support groups in Denmark and Ireland. Although some participants found it difficult to participate in a peer support group, most participants described them as safe and supportive spaces that aided in their grief process. Griffin and colleagues [32] surveyed participants of peer-led support groups and found a significant improvement in wellbeing and grief reactions, adjusting for time since bereavement. The participants reported that the groups provided a sense of belonging and hope. This study underscored the enduring mental health challenges for those bereaved by suicide and the need for the ongoing availability of peer-led support in postvention.

In conclusion, the studies included in this Special Issue emphasize the short- and long-term impact of suicide loss, the needs of those bereaved by suicide, and appropriate interventions in the aftermath of suicide loss. The included studies showed that suicide bereavement impact various populations, far beyond the nuclear family of the deceased. Moreover, these studies highlight the risk of long-term mental health challenges and adverse grief reactions among those bereaved by suicide. Health professionals, often involved in suicide prevention, should not be overlooked with regard to support after a loss by suicide.

## 4. Public Health Approach to Postvention

As individuals and population groups may experience suicide loss differently, and their needs for support may evolve over time, various support options must be available. A recent review of the literature indicates that adjusting the support to the level of the impact of the bereavement, and focusing the support on the grief rather than on other outcomes are key characteristics of effective suicide bereavement support [33]. As indicated by studies on peer-led support in this Special Issue, the involvement of trained peers who may serve as role models or community members enhancing the social support network, may contribute to the effectiveness of suicide bereavement support [33].

According to the public health model, universal interventions target those who experience little impact from a death by suicide. Such interventions may include, for example, leaflets, fact sheets, or online information about suicide bereavement for wide audiences. Selective interventions target people with moderate grief reactions and include support groups, community and educational support, and training for health professionals. Indicated interventions, focusing on people with adverse grief and mental health reactions, may include specific grief therapy, and psychological and psychiatric support [33]. Adopting a public health approach to postvention can help to better tailor service delivery to the needs of the bereaved individuals and to align postvention with suicide prevention programs, which are also often based on this public health model. The training of service providers, including peers, volunteers, and health professionals, is paramount. Further evaluation and research will strengthen the evidence of effectiveness of the interventions and service delivery in this important field.
